# The dataset of duplex ultrasound assessment of the internal mammary artery in women with unilateral mastectomy followed by radiotherapy for breast cancer

**DOI:** 10.1016/j.dib.2023.110004

**Published:** 2023-12-22

**Authors:** Thanh-Phong Le, Anh T. Le, Tan N.D. Huynh, Thu-Ha Dao, Pascal Desgranges, Romain Bosc

**Affiliations:** a Department of Vascular Surgery, Cho Ray Hospital, Ho Chi Minh City, Viet Nam; b Oncology Center, Cho Ray Hospital, Ho Chi Minh City, Viet Nam; c Department of Diagnostic Ultrasonography, Cho Ray Hospital, Ho Chi Minh City, Viet Nam; d Department of Imaging, Henri Mondor Hospital, Creteil, France; e Department of Vascular Surgery, Henri Mondor Hospital, Creteil, France; f Department of Plastic, Reconstructive and Aesthetic Surgery, Intercity Hospital of Creteil, Paris, France; g Private Peupliers Hospital, Ramsay, 8, Place de l'Abbé Georges Henocque, Paris, France; h University of Paris-Est, Creteil, France

**Keywords:** Internal mammary artery, Artery diameter, Blood flow, TAMAX, TAMEAN, Radiotherapy, Breast cancer, Microsurgery

## Abstract

Adjuvant radiotherapy for breast cancer may involve some incidental exposure of the ipsilateral internal mammary artery to ionizing radiation. However, the relevant evidence is limited and inconsistent. The dataset presented in this article contains the information used to assess the effects of accidental radiation exposure on the internal mammary artery in patients with unilateral total mastectomy followed radiotherapy for breast cancer. The study population consists of two groups: the irradiated group and the control group. The left and right internal mammary arteries were assessed through the second intercostal spaces using a computed sonography system (Vivid S6; GE, Tirat Carmel, Israel) equipped with a 5.5 - 11 MHz transducer. The recorded parameters were the diameter, time-averaged maximum velocity, and blood flow of the internal mammary artery.

The dataset contains two files of data: a raw and an analyzed data. The raw data file contains the individual information of each participant, including demographic characteristics and the parameters of the internal mammary artery duplex ultrasound imaging. The analyzed data file was made up of R Markdown, a markup language of R. The results of data analysis were presented in the related research article which has been accepted for publication in the Annals of Vascular Surgery. The dataset presented in this article may be reused for further studies in which the internal mammary artery is considered as potential donor or recipient vessels for a vascular bypass or free flap anastomosis.

Specifications Table

**Table:** 

Subject	Health and medical sciences: Cardiology and Cardiovascular Medicine
Specific subject area	Duplex ultrasound assessment of the internal mammary artery in patients with unilateral mastectomy followed by radiotherapy for breast cancer.
Data format	Raw and analyzed
Type of data	Table, code
Data collection	Data were acquired from a prospective database that comprises all transthoracic duplex ultrasound of the IMA performed at the Imaging Diagnostic Department of Cho-Ray hospital, using a computed sonography system (Vivid S6; GE, Tirat Carmel, Israel) equipped with a 5.5 - 11 MHz transducer.
Data source location	Cardiovascular Center, Oncology Center, Imaging Diagnostic Department, Cho-Ray Hospital, Ho Chi Minh city 72713, Vietnam
Data accessibility	Repository name: zenodo.org Data identification number: https:/zenodo.org/records/10077206 Direct URL to data: https:/doi.org/10.5281/zenodo.10077206
Related research article	The manuscript supports a related research article which was submitted to the Annals of Vascular Surgery: AVS-D-23-00926 and accepted for publication. Duplex Imaging Assessment of the Internal Mammary Arteries in Women after Unilateral Mastectomy and Radiotherapy for Breast Cancer Thanh-Phong Le, M.D., Anh T. Le, M.D., Ph.D., Tan N. D. Huynh, M.D., Khanh Q. Huynh, M.D., Ph.D., Thu-Ha Dao, M.D., Pascal Desgranges, M.D., Ph.D., Romain Bosc, M.D., Ph.D.

## 1 Value of the Data

• The evidence concerning the effects of incidental radiation exposure on the internal mammary artery (IMA) is limited and inconsistent. In fact, only two articles with different designs were reported in the literature with conflicting results. One study, using Duplex ultrasound to compare the blood flow of the irradiated and non-irradiated IMAs, found no significant difference between two groups; while another using computed tomographic angiography to compare the diameter of the irradiated and non-irradiated IMAs in patients with mastectomy followed by radiation therapy demonstrated a significant difference.
• The IMA plays an essential role in free-flap breast reconstruction (FFBR) after mastectomy for breast cancer. This vessel may also be a potential conduit for coronary artery bypass grafting (CABG) due to an increased risk of radiation-induced coronary diseases in this population. The relevance of these data may be appreciated in the context of an increasing need for FFBR after total mastectomy for breast cancer in which the irradiated IMA is commonly chosen as a recipient vessel. Similarly, the quality of the irradiated IMA can be preoperatively assessed before a CABG.
• These data concerning to the diameter and blood flow measurements using by Duplex ultrasound in the Asian irradiated and non-irradiated population may be reused by other researchers. The significant differences between the irradiated and non-irradiated IMAs with regard to the diameter and blood flow may be reused for further studies in which the irradiated IMAs would be considered as a recipient vessel.

## 2 Background

The evidence relating to the effects of accidental radiation exposure on the IMA in patients with mastectomy followed by radiotherapy is limited and inconsistent. We hypothesized that there would be effects of incidental radiation exposure on IMAs. Theoretically, there were several means to test the hypothesis: catheter-based arteriography (CBA), computed tomographic angiography (CTA), and duplex ultrasound. However, CBA and CTA may be not suitable for a large-scale prospective study due to the high cost, renal toxicity, radiation exposure. Duplex ultrasound imaging (DUS), on the contrary, has been appreciated as a non-invasive, inexpensive, and reliable means to assess the IMA. In this setting, the data was collected using DUS for assessment of the diameter, and blood flow of the concerned IMAs. This data article supplies more detailed information which was not presented in the related research article published on the Annals of Vascular Surgery.

## 3 Data Description

This article describes the dataset of the duplex ultrasound evaluation of the IMA both in patients with unilateral mastectomy followed by radiotherapy for breast cancer and in the non-irradiated control patients. The dataset [1] contains two files of data: a raw and an analyzed data as follows.

**The raw data file** contains the individual information of each participant, including demographic characteristics and the parameters of the IMA duplex ultrasound imaging. Recorded variables are those presented in Tables 1 and 2 and individual values can be consulted on the accompanying file.

**Table 1:** Baseline demographic characteristics recorded in the dataset.

Variables	Unit	Dataset variable name
Radiation therapy	(0: no, 1: yes)	rx
Age	year	age
Body mass index	Kg/m^2^	bmi
Hypertension	(0: no, 1: yes)	hbp
Coronary artery disease	(0: no, 1: yes)	cad
Smoking	(0: no, 1: yes)	smoking
Diabetes	(0: no, 1: yes)	diabetes
Date of radiation completion	mm-dd-yyyy	datex
Date of duplex performance	mm-dd-yyyy	datep
Time from radiotherapy completion	day	days

**Table 2:** Ultrasonographic parameters of the IMA recorded in the dataset.

Variables	Unit	Dataset variable name
Mastectomy location	(0: left, 1: right)	m.side
Left IMA diameter	cm	l.dia
Left IMA TAMAX	ml/sec	l.tamax
Left IMA blood flow	ml/ min	l.bf
Right IMA diameter	cm	r.dia
Right IMA TAMAX	ml/sec	r.tamax
Right IMA blood flow	ml/min	r.bf

Table 1 specifies baseline demographic characteristics, including age, body mass index (BMI), cardiovascular risk factors (hypertension, coronary artery disease, smoking, diabetes) as well as the date of radiation completion, the date of duplex ultrasound investigation, and the time from radiotherapy completion.

Table 2 presents the location of total mastectomy, and the ultrasonographic parameters of the left and right IMAs (diameter, time averaged maximum velocity (TAMAX), blood flow).

The analyzed data file are made up of R code in type of R Markdown language, using R version 4.1.1 for Windows (IMA.Rmd) [2]. The diameter and blood flow of the irradiated IMAs regardless of laterality were compared to those of the non-irradiated contralateral IMAs in the irradiated group as well as in the control group, using nonparametric Wilcoxon rank-sum test. The results of the data analysis were mainly presented in the related research article that has been accepted for publication in the Annals of Vascular Surgery.

## 4 Experimental Design, Materials and Methods

### 4.1 The selection criteria

From November 2021 to December 2022, all scheduled follow-up patients with a history of total mastectomy followed by radiation therapy were invited to participate the study if they did not meet the following criteria: (1) under 18 years of age, (2) partial mastectomy, (3) bilateral breast cancer, (4) additional chest wall radiation for reasons different from breast cancer, (5) concomitant vascular diseases possibly leading to changes in IMA's blood flow, (6) incompleted adjuvant radiotherapy.

In the same period, the control group was selected from the women presenting with a breast condition at the Oncology Center of Cho-Ray hospital according to the following criteria: (1) over 18 years of age, (2) without a history of chest-wall radiotherapy for any reasons, (3) without a history of cardiovascular diseases probably resulting in a reduced diameter or blood flow of IMAs.

### 4.2 Sample size estimation

According to a previously published study we considered that the mean (SD) BF of the IMAs would be 36 (8.3) ml/m in the irradiated group and 39 (7.2) ml/m in the non-irradiated group. Based on 0.8 power to detect a significant difference (p = 0.05, two-sided), 106 people were required for each study group. To compensate for unevaluable people, more than 116 participants were planned to enrol for each group.

### 4.3 Duplex imaging protocol

With the patient in the supine position, the left and right IMAs were assessed through the second intercostal spaces using a computed sonography system (Vivid S6; GE, Tirat Carmel, Israel) equipped with a 5.5 - 11 MHz transducer. The recorded ultrasonographic parameters were the diameter, TAMAX, and blood flow.

The blood flow was calculated by the equation: blood flow = Cross-sectional Area (A) x TAMAX x 60. The A is obtained as πr^2^ (or its equivalent, D x 0.785), where r represents one half of the IMA diameter (D) (centimeters).

### 4.4 Statistical analysis

All values were expressed as mean ± standard deviation (SD) or median and interquartile range. T test was used for continuous data with normal distribution while continuous data without normal distribution was analyzed using nonparametric Wilcoxon rank-sum test. The Spearman R test was used to assess the association between time from radiotherapy completion and the irradiated IMAs diameter or blood volume.

The primary outcomes of this study were the differences in diameter and in blood flow between the irradiated IMAs and non-irradiated IMAs, the secondary outcome was the correlation between post-radiation time and the irradiated IMA diameter or blood flow. All statistical analyses were performed using R, version R 4.1.1, for Windows [3].

## Limitations

Not applicable

## Ethics Statement

The research was carried out in accordance with the Declaration of Helsinki and was approved by the local Ethics Committee (Cho-Ray Hospital, approved March 24th, 2021, N^0^ 1144/GCN-HĐĐĐ). All participants obtained the informed consent and were anonymized in all data.

## CRediT authorship contribution statement

**Thanh-Phong Le:** Conceptualization, Methodology, Software, Writing – review & editing. **Anh T. Le:** Methodology, Writing – review & editing. **Tan N.D. Huynh:** Data curation. **Thu-Ha Dao:** Methodology, Writing – review & editing. **Pascal Desgranges:** Conceptualization, Methodology, Writing – review & editing. **Romain Bosc:** Conceptualization, Methodology, Writing – review & editing.

## Declaration of Competing Interest

The authors declare that they have no known competing financial interests or personal relationships that could have appeared to influence the work reported in this paper.

## Data Availability

The dataset of duplex ultrasound assessment of the IMA in women with unilateral mastectomy followed by radiotherapy for breast cancer (Original data) (zenodo.org)
